# Periodontitis of maxillary teeth screened by community periodontal index is associated with chronic rhinosinusitis defined by EPOS 2020 guideline

**DOI:** 10.1038/s41598-023-43474-3

**Published:** 2023-10-18

**Authors:** Jiwon Kwak, Munsoo Han, Yujin Jeong, Bo Yoon Choi, Dabin Lee, Sang Hag Lee, Tae Hoon Kim

**Affiliations:** 1 Department of Otorhinolaryngology-Head and Neck Surgery, College of Medicine, Korea University, 73, Goryeodae-Ro, Seongbuk-Gu, Seoul, 02841 Republic of Korea; 2 Mucosal Immunology Institute, College of Medicine, Korea University, Seoul, Republic of Korea; 3 Department of Biostatistics, Korea University College of Medicine, Seoul, Republic of Korea; 4 Department of Otorhinolaryngology-Head and Neck Surgery, Konkuk University School of Medicine, Seoul, Republic of Korea

**Keywords:** Epidemiology, Medical research

## Abstract

We aimed to evaluate the association between periodontitis in the upper jaw and chronic rhinosinusitis (CRS) using the nationwide Korean National Health and Nutrition Examination Survey (KNHANES) data. In this cross-sectional study, data of KNHANES participants enrolled between 2008 and 2012 were reviewed. Periodontitis of the upper teeth was diagnosed by dentists according to the community periodontal index with standardized methods. CRS was diagnosed by otorhinolaryngologists according to the European Position Paper on Rhinosinusitis and Nasal Polyps 2020 with nasal endoscopy findings. We also evaluated the association between periodontitis and CRS according to smoking and drinking status. Univariate and multivariate logistic regression analyses were performed. Overall, 28,761 participants were eligible for analysis, and 210 were diagnosed with CRS. Periodontitis was associated with CRS diagnosis (odds ratio [OR] = 1.391, 95% confidence interval [CI] = 1.013–1.912). Non-drinkers showed no significant association between periodontitis and CRS (OR = 1.142, 95% CI 0.746–1.749). However, among drinkers, periodontitis was significantly associated with CRS (OR = 1.733, 95% CI 1.091–2.753). The number of smokers with CRS was not statistically sufficient and a logistic regression model based on smoking status could not be generated. Individuals with periodontitis in the upper jaw may need to consult an otorhinolaryngologist for comorbid CRS especially according to drinking status.

## Introduction

Chronic rhinosinusitis (CRS) is defined as an inflammatory disease of the paranasal sinuses that lasts for more than 12 weeks^1^. The typical clinical manifestations of CRS include nasal discharge, nasal congestion, pain or sense of fullness around the cheeks or forehead, and reduction or loss of smell^2^. According to recent studies, CRS is prevalent in approximately 10% of the general population, impacting quality of life and increasing the burden of healthcare costs worldwide^3–5^. CRS is primarily treated with medications, and in case of failure of drug interventions, surgical treatment is considered^6^.

Numerous factors contribute to the pathophysiology of CRS, including host-related and environmental factors^7^. Previous studies have shown that smoking induces chronic irritation of the upper respiratory epithelium, which is suggestive of its adverse influence on CRS^8^. Additionally, patients with CRS who have nasal polyps have a high tendency to present with hypersensitivity to alcohol^9^. Since the etiology of CRS is multifactorial, some patients have persistent or recurrent disease even after aggressive treatment^10^. Therefore, understanding the pathophysiology of this disease and identifying any relevant intrinsic or extrinsic risk factors are important.

Odontogenic sinusitis accounts for approximately 10% of all CRS cases, most of which are caused by iatrogenic trauma during dental surgery^11^. In addition to the direct spread of inflammation, as observed in dental implant procedures, chronic periodontitis can also cause CRS through an increase in systemic inflammatory markers^12,13^. Previous epidemiological studies that have analyzed the correlation between CRS and periodontitis using Korean National Health Insurance Service-Health Screening Cohort data have the following limitation: periodontitis and CRS have been defined on the basis of inconsistent diagnostic measures and by different physicians^12,14^.

Therefore, in this study, we investigated the correlation between periodontitis and CRS using the Community Periodontal Index (CPI), which was recorded by dentists. To diagnose CRS, otorhinolaryngologists examined the symptoms and endoscopic findings of CRS in each participant. In addition, since smoking and drinking habits are associated with a greater prevalence of inflammatory diseases in patients with periodontitis, we further analyzed the relationship between periodontitis and CRS among smokers and non-smokers and among drinkers and non-drinkers^15^.

## Material and methods

### Study population

In this study, data from the Korean National Health and Nutrition Examination Survey (KNHANES) from 2008 to 2012 were used. The KNHANES is a nationwide survey conducted annually in South Korea by the Korea Centers for Disease Control and Prevention to analyze current trends in the health and nutritional status of the population. On the basis of the national census data, approximately 10,000 people were enrolled to represent the Korean population using a random sampling method. They were interviewed, filled out questionnaires on their nutritional and health status, and underwent physical examinations by healthcare providers. The KNHANES survey results are posted on the website every year and can be accessed and used for academic research. Between 2008 and 2012, 392 sampling survey districts were extracted, and 8400 household members participated in the survey. Health surveys and examinations were conducted at the mobile examination center, and nutrition surveys were conducted through direct visits to the target households.

Between 2008 and 2012, 150 otorhinolaryngologists from 47 institutions recorded detailed medical interviews and endoscopic findings. Since objective diagnostic findings, such as endoscopy findings, are essential for diagnosing CRS, we used the data from the above-mentioned period in this study. Participants who are 18 years old or more were enrolled for the study.

### Diagnosis of chronic rhinosinusitis according to the European Position Paper on Rhinosinusitis and Nasal Polyps 2020 guidelines

We followed the European Position Paper on Rhinosinusitis and Nasal Polyps (EPOS) 2020 guidelines as diagnostic criteria when selecting the participants for the CRS group. According to these guidelines, two of the following four symptoms must be present for diagnosing CRS: nasal obstruction, rhinorrhea, facial pain/pressure, and loss of smell. In addition, inflammation of the sinuses must be proven through objective findings using nasal endoscopy. From 2008 to 2012, additional analyses using nasal endoscopy were performed in the KNHANES by otorhinolaryngologists.

### Dental examination

Dental examination was performed by public health dentists, who measured the depth of the periodontal pocket using a periodontal probe with a 0.5 mm ball tip. They scored the dental health status of each individual using the CPI, which is an indicator of periodontal disease severity. Scores are assigned on the basis of the following five criteria: healthy periodontal tissue (CPI 0), gingival bleeding on probing (CPI 1), presence of tartar (CPI 2), and depth of periodontal pocket (probing depth of 4–5 mm indicates CPI 3 and that of ≥ 6 mm indicates CPI 4). CPI scores of 1 and 2 indicate gingivitis, and those of 3 and 4 indicate periodontitis, which require more aggressive treatment. In the KNHANES, CPI 3 and 4 groups were defined as having periodontal diseases. Examiners checked the molars and premolars of the upper and lower jaws on the left side to assign scores. The right side examination was conducted with the same method. We excluded the data of central incisor, lateral incisor and canine teeth. Our study specifically analyzed the correlation between periodontitis in upper teeth and CRS; therefore, in this manuscript, the term “CPI positive” will be used to indicate CPI scores 3 or 4 in the upper teeth, regardless of the presence of periodontitis in the lower teeth.

### Statistical analyses

In this study, the analysis was conducted using Statistical Analysis System version 9.4 (SAS Institute, Inc., Cary, NC, USA). The baseline characteristics of the participants included age, sex, income, education level, occupation, drinking, smoking, residence, obesity, hypertension, and diabetes mellitus; these were used to adjust for confounding factors. These characteristics were analyzed using chi-square tests and one-way analyses of variance to determine the differences between the normal and CRS groups. Logistic regression analyses were performed to estimate the association between variables, including the odds ratio (OR) and 95% confidence interval, and the significance level was set at *p* < 0.05. The confounding variables were adjusted using multivariable logistic regression analysis to exclude the potential relevant variables. The significance level of the multivariable analysis was set at *p* < 0.25.

As a secondary analysis, we created drinker and non-drinker subgroups, as well as smoker and non-smoker subgroups, and analyzed the correlation between the CPI score and CRS in each group.

### Ethical considerations

All methods in this study was conducted according to the guidelines laid down in the Declaration of Helsinki. The KNHANES has been reviewed and approved annually by the Research Ethics Review Committee of the KCDC (IRB No. 2008-04EXP-01-C, 2009-01CON-03-2C, 2010-02CON-21-C, 2011-02CON-06-C, and 2012-01EXP-01-2C). Written informed consent was obtained from all participants of the KNHANES.

### Ethics approval and consent to participate

This study is based on the previously conducted research data of National Research Foundation of Korea which are open to the public. Therefore, the ethical approval and patient consent were not needed in this study.

## Results

### Association between CRS and CPI scores

**Figure 1:**
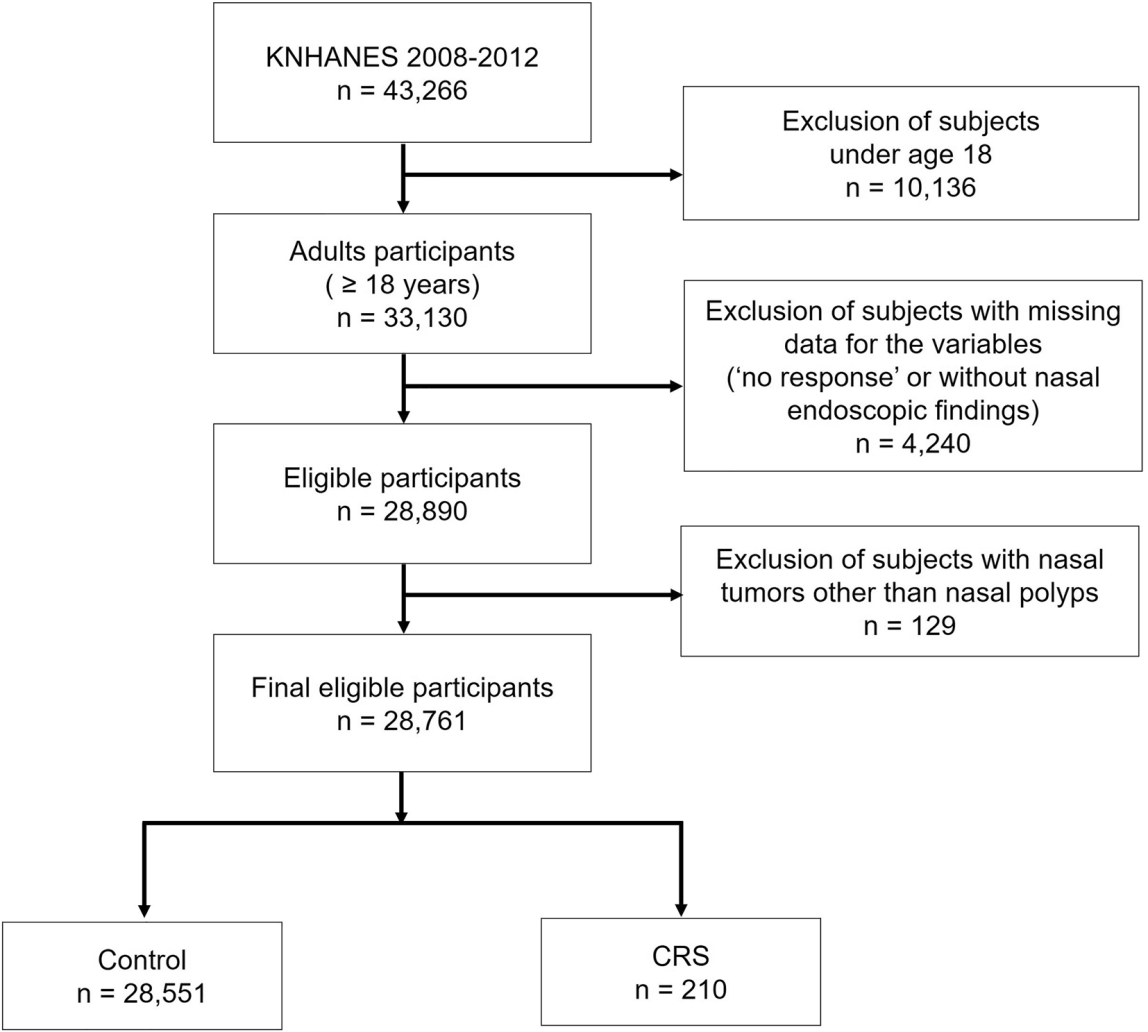
Flowchart of eligible participants. *KNHANES* Korean National Health and Nutrition Examination Survey, *CRS* chronic rhinosinusitis.

Of 43,266 participants in the KNHANES study between 2008 and 2012, only 28,761 were eligible for inclusion after excluding participants aged < 18 years, with missing data, or with a nasal mass or tumor according to endoscopic findings. Of the final 28,761 participants, 28,551 were in the control group, and 210 (0.73%) met the criteria of the EPOS 2020 guidelines for CRS (Fig. 1).

The minimum sample size required for the logistic regression analysis used in this study was calculated using the G*power program, based on the variable with the smallest effect size among the factors influencing periodontitis in the previous study^16^. With a two-tailed test, where OR = 1.22, R^2^ other X = 0.7, significance level α = 0.05, and power of 0.80, the minimum sample size was 4794 individuals. Among the subjects in the KNHANES, the final number of participants who met the inclusion criteria was 28,761, which is sufficiently larger than the calculated minimum sample size.

The baseline characteristics of all participants included basic information for each group, such as age, sex, education level, occupation, and residence, in addition to obesity, hypertension, diabetes, drinking, and smoking (Table 1). In the univariate analysis, age, sex, income, education, drinking, smoking, residency, and CPI showed significant differences (*p* < 0.25). These variables were analyzed using multivariable logistic regression. In this analysis, age, sex, income, education level, residence, drinking, and smoking were not significantly different between the normal and CRS groups. Only the proportion of patients with CRS with CPI positivity was significantly higher than that of the normal group (*p* < 0.05) (Table 2).

**Table 1:** Baseline characteristics of participants according to diagnosis of chronic rhinosinusitis (CRS).

	Study participants		*p* value
	Control (n = 28,551)	CRS (n = 210)	
Age, years (mean ± SD)	49.7 ± 16.79	54.5 ± 16.97	< .0001
Sex, n (%)			0.0695
Male	12,227 (42.83)	103 (49.05)	
Female	16,324 (57.17)	107 (50.95)	
Residence, n (%)			0.0059
Urban	22,006 (77.08)	145 (69.05)	
Rural	6,545 (22.92)	65 (30.95)	
Education level, n (%)			0.0006
≤ Elementary school	7454 (26.58)	75 (36.59)	
Middle school	3251 (11.59)	31 (15.12)	
High school	9543 (34.02)	61 (29.76)	
≥ College or graduate school	7800 (27.81)	38 (18.54)	
Household income, n (%)			0.005
Lower	5739 (20.38)	54 (26.21)	
Lower middle	7079 (25.14)	65 (31.55)	
Upper middle	7703 (27.36)	40 (19.42)	
Upper	7634 (27.11)	47 (22.82)	
Occupation, n (%)			0.4715
Managers, experts and related workers	3292 (11.77)	17 (8.29)	
Office workers	2157 (7.71)	15 (7.32)	
Service and sales personnel	3497 (12.5)	24 (11.71)	
Agricultural, forestry and fishery skilled workers	2467 (8.82)	25 (12.2)	
Technicians, equipment and machine operation and assembly workers	2527 (9.03)	16 (7.8)	
Simple labor workers	2421 (8.65)	20 (9.76)	
Unemployed (housewife, student, etc.)	11,615 (41.52)	88 (42.93)	
Drinking, n (%)			0.0322
Never	13,124 (47.4)	111 (54.95)	
Yes	14,566 (52.6)	91 (45.05)	
Smoking, n (%)			0.3417
None (never-smoker or ex-smoker)	21,894 (79.08)	155 (76.35)	
Yes (current smoker)	5792 (20.92)	48 (23.65)	
Obesity, n (%)			0.5304
Low weight (BMI < 18.5)	1326 (4.73)	7 (3.43)	
Normal (18.5 ≤ BMI < 25)	17,852 (63.69)	127 (62.25)	
Obese (25 ≤ BMI)	8852 (31.58)	70 (34.31)	
Hypertension, n (%)			0.1281
No	22,317 (78.17)	155 (73.81)	
Yes	6234 (21.83)	55 (26.19)	
Diabetes, n (%)			0.2663
No	26,290 (92.08)	189 (90)	
Yes	2261 (7.92)	21 (10)	
CPI, n (%)			< .0001
No (0,1,2)	20,389 (71.41)	121 (57.62)	
Yes (3,4)	8162 (28.59)	89 (42.38)	

*CRS* chronic rhinosinusitis, *SD* standard deviation, *BMI* body mass index, *CPI* community periodontal index.

**Table 2:** Univariable and multivariable logistic regression analyses of the risk factors for chronic rhinosinusitis (CRS).

	Univariable		Multivariable	
	OR (95% CI)	*p* value	OR (95% CI)	*p* value
Age	1.017 (1.009–1.026)	< .0001*	1.005 (0.992–1.018)	0.4392
Sex				
Male	1 (reference)		1 (reference)	
Female	0.778 (0.593–1.021)	0.0701*	0.747 (0.533–1.048)	0.0909
Household income, n (%)		0.0056*		0.1598
Lower	1 (reference)		1 (reference)	
Lower middle	0.976 (0.679–1.402)	0.8949	1.22 (0.826–1.804)	0.3179
Upper middle	0.552 (0.366–0.832)	0.0045	0.764 (0.482–1.211)	0.2521
Upper	0.654 (0.442–0.969)	0.0342	0.947 (0.6–1.494)	0.814
Education level, n (%)		0.0008*		
≤ Elementary school	1 (reference)		1 (reference)	
Middle school	0.948 (0.622–1.443)	0.8023	1.095 (0.693–1.733)	0.6968
High school	0.635 (0.453–0.892)	0.0088	0.848 (0.538–1.338)	0.4782
≥ College or graduate school	0.484 (0.327–0.716)	0.0003	0.731 (0.43–1.243)	0.2477
Drinking	0.739 (0.559–0.976)	0.0328*	0.779 (0.568–1.069)	0.1215
Residency				
Urban	1 (reference)		1 (reference)	
Rural	1.507 (1.123–2.022)	0.0062*	1.198 (0.871–1.648)	0.2675
CPI, n (%)				
No (0,1,2)	1 (reference)		1 (reference)	
Yes (3,4)	1.837 (1.396–2.419)	< .0001*	1.391 (1.013–1.912)	**0.0417**

In multivariable logistic regression analysis, confounding variables with *p* < 0.25 in univariable logistic regression analysis are adjusted (age, sex, household income, education level, drinking, residency, and CPI). Variables with asterisk indicate *p* values < 0.25. Variable with bold indicate *p* values < 0.05.

*CRS* chronic rhinosinusitis, *CPI* newly diagnosed community periodontal index, *OR* odds ratio, *CI* confidence interval.

### Alcohol and smoking

The participants were divided into drinking and non-drinking groups, with 13,225 drinkers and 14,657 non-drinkers. In the drinking group, 91 patients met the criteria for CRS diagnosis, and 14,566 were classified as normal. Among these participants, we analyzed the ORs of age, sex, income, residency, obesity, hypertension diagnosis, and CPI in the CRS and normal groups. Only the CPI was significantly higher in the CRS group than in the normal group, with an OR of 1.733 (95% confidence interval = 1.091–2.753) (*p* = 0.02) (Table 3). Using the same method, in the non-drinking group, the subgroup of CRS had 111 participants, and the normal group had 13,124 participants. The analysis items were income, education, smoking, residence, hypertension diagnosis, and CPI. Unlike in the drinking group, the difference in the CPI between the two subgroups was not statistically significant among non-drinkers.

**Table 3:** Multivariable logistic regression analysis between drinking and the prevalence of periodontitis in patients with chronic rhinosinusitis (CRS).

	Non-drinking		Drinking	
	OR (95% CI)	*p* value	OR (95% CI)	*p* value
CPI, n (%)				
No (0,1,2)	1 (reference)		1 (reference)	
Yes (3,4)	1.142 (0.746–1.749)	0.5406	1.733 (1.091–2.753)	0.0200*

Variables with asterisk indicate *p* values < 0.05.

*CRS* chronic rhinosinusitis, *CPI* newly diagnosed community periodontal index, *OR* odds ratio, *CI* confidence interval.

We also divided the patients into smoker and non-smoker groups; 5840 participants were smokers, and 22,049 were non-smokers. Among the smoker group, 48 satisfied the diagnostic criteria of CRS, and this number was too small to be modeled; therefore, statistical analysis was not possible.

## Discussion

Among 28,761 participants of the KNHANES from 2008 to 2012, periodontitis of the upper jaw was significantly associated with CRS, showing a high OR in the multivariable logistic regression analysis. After analyzing 14,657 participants, this tendency was found to be more prominent among the drinker group than among the non-drinker group. The association between CRS and periodontitis among the smokers was not statistically analyzed owing to the small sample size.

This is the first study on CRS conducted using the nationwide large-scale KNHANES, which represents the health and nutritional status of the general Korean population^17^. Although the correlation between CRS and periodontitis has been analyzed before, this study is meaningful because both dentists and otorhinolaryngologists were involved in the diagnosis of the participant’s current status, and it was not dependent on the self-questionnaire or diagnostic codes. Therefore, the participants of this study were diagnosed by specialists, and the diagnostic criteria for CRS were based on the EPOS 2020 guidelines.

Odontogenic sinusitis is an inflammatory disease of the sinonasal mucosa induced by dental pathology, and it often results from previous dental procedures or trauma that induces maxillary sinus inflammation^11^. The most common cause of odontogenic CRS is iatrogenic injury of the mucoperiosteum or Schneiderian membrane, which allows inflammation to mechanically spread to the maxillary sinus^18^. This etiology accounts for approximately 65.7% of odontogenic sinusitis cases^19^. Hence, odontogenic sinusitis has been the focus when examining the effects of iatrogenic trauma in the upper tooth region on CRS development. We hypothesized that not only direct trauma from iatrogenic dental procedures, but also the spread of inflammation from periodontitis can indirectly induce microscopic structural changes in the Schneiderian membrane. Since the participants with periodontitis in this study included those with dental inflammation and not just those with iatrogenic damage, the findings suggest that periodontitis unassociated with iatrogenic injury could also affect the occurrence of CRS.

The inflammatory condition of the bony compartment could explain the association between CRS and periodontitis. Osteomyelitis is an inflammatory condition of the bone, beginning in the medullary cavity and extending to the bony cortex. Patients with chronic periodontitis have a high tendency to develop osteomyelitis^20,21^. The proposed etiology is the biofilm produced by oral anaerobes, which can even influence inflammation in distant bones, such as the femur, through the blood stream^22^. Moreover, periodontitis can induce pathological changes in tissues adjacent to the oral cavity, such as the upper jaws, with great ease, leading to chronic inflammation of the paranasal sinuses.

Anatomical proximity may also explain the association between CRS and periodontitis. A similar mechanism can be found in deep neck infection, which is characterized by cellulitis or abscess formation in the cervical spaces and fascial plane^23^. The most common cause of deep neck infection is dental infection, which spreads along the connective tissue from the oral cavity to the adjacent cervical spaces^24^. Similarly, both the maxillary gingiva and sinus are in proximity to the maxillary bone, enabling the easy spread of inflammation from one structure to the other through the maxillary bone. We speculate that the commonality of inflammatory conditions and anatomical proximity may have created a link between CRS and periodontitis.

In a previous study, chronic alcohol consumption was found to have systemic effects and to induce multifocal osteonecrosis^25^. Osteonecrosis refers to the destruction of bony compartments, such as bone cells and marrow, leading to a prominent chronic inflammatory state^26^. According to some studies, osteonecrosis induces inflammation of the synovial membrane, activating immune cells in the joints, even in patients without confounding symptoms of arthritis^27^. Since the oral mucosa of the upper jaw comes in direct contact with alcohol, necrotic changes in the bony structure or mucosa, including the Schneiderian membrane, are more likely to occur. Alcohol-induced necrotic changes in the maxilla may cause an inflammatory response like CRS, which is similar to the pathogenesis of arthritis, thereby inducing immune cell responses in the joints of patients with systemic osteonecrosis.

Meanwhile, although there was no independent contribution in the multivariable logistic regression analyses, residence, education level, and household income showed statistically significant difference between the participants with CRS and control in the baseline characteristics analysis. In the international guideline the aggravating or contributing effect of the demographic factors were reviewed and it has been stated that the causal link cannot be clearly established^28^. As for our data from the KNHANES, the effect of atmospheric exposure could have been more significant for participants who live in rural area than urban area. Several studies reported that air pollutants such as particulate matter with aerodynamic diameters ≤ 10 µm (PM_10_) in the atmosphere has association with CRS^29,30^. From the results of this study, it is rather difficult to define the exact causal relationship between CRS and the baseline demographic factors. However, the multivariable regression analysis showed that after correction of the other factors, CPI was the independent factor associated with CRS, and it can be assumed that worse periodontal status could have been related to rural area residency, lower household income, or lower education level.

A limitation of this study is that a causal relationship between CRS and periodontitis could not be identified owing to the cross-sectional design. Further, KNHANES data from only 2008 to 2012 include the endoscopic findings of otorhinolaryngologists. After 2013, no endoscopic findings are available, which prevents analysis of the current trend of the relationship between CRS and CPI. Moreover, although specialists’ endoscopic findings were used to diagnose CRS, some patients may have had polyps or discharge in the posterior part of the nasal cavity (for example, the posterior ethmoid sinus or sphenoid sinus), which are difficult to detect and may have remained undiagnosed. Computed tomography of the paranasal sinuses could help identify participants with CRS more accurately. Finally, CPI measurements are affected by the pressure applied during probing and by inter-examiner variability (approximately 66% for sensitivity and 85% for specificity)^31^, which may have contributed to errors, considering that KNHANES is a large-scale study. Another limitation regarding dental examination is that CPI was not graded for each tooth but according to the four areas of oral cavity; upper right, upper left, lower right, and lower left.

## Conclusions

We analyzed data of 28,761 KNHANES participants enrolled between 2008 and 2012 using multivariable logistic regression. In this study, CRS was diagnosed using the EPOS 2020 guidelines, considering patients’ symptoms and endoscopic findings, and periodontitis was defined on the basis of CPI scores. These results demonstrated a significant association between CRS and periodontitis. We also divided the patients into subgroups according to their smoking and drinking status. Among drinkers, the CPI was significantly high in patients with CRS, while CPI and CRS show no statistically significant association among non-drinkers. Meanwhile, the subgroup of smokers was not analyzed because of the small sample size. Therefore, even in the absence of a history of invasive dental procedures, periodontitis could influence the development of CRS; this indicates that appropriate oral hygiene care might lower the incidence of CRS.

## Data Availability

The KNHANES data are open to the public; therefore, any researcher can obtain the raw data by requesting it on the website (https://knhanes.kdca.go.kr/knhanes/eng/index.do).

## Abbreviations

CRS: Chronic rhinosinusitis
CPI: Community periodontal index
EPOS: European Position Paper on Rhinosinusitis and Nasal Polyps
KNHANES: Korean National Health and Nutrition Examination Survey
CI: Confidence interval
OR: Odds ratio
